# Prognostic and predictive significance of VEGF, CD31, and Ang-1 in patients with metastatic clear cell renal cell carcinoma treated with first-line sunitinib

**DOI:** 10.17305/bjbms.2022.7675

**Published:** 2023-01-06

**Authors:** Marija Kraljević, Inga Marijanović, Maja Barbarić, Emir Sokolović, Merima Bukva, Timur Cerić, Teo Buhovac

**Affiliations:** 1 Oncology Clinic, University Clinical Hospital Mostar, Mostar, Bosnia and Herzegovina; 2 Laboratory of Morphology, Department of Histology and Embryology, School of Medicine, University of Mostar, Mostar, Bosnia and Herzegovina; 3 Clinic of Oncology, Clinical Center University of Sarajevo, Sarajevo, Bosnia and Herzegovina; 4 Association of Basic Medical Sciences of FBIH, Sarajevo, Bosnia and Herzegovina

**Keywords:** Carcinoma, renal cell, sunitinib, VEGF-A, Angiopoietin-1, Platelet Endothelial Adhesion Molecule 1, Cluster of differentiation 31, microvascular density, survival rate, progression-free survival, fluorescent antibody technique

## Abstract

The most common type of renal cell carcinoma (RCC) is clear cell renal cell carcinoma (ccRCC), which has a high metastatic potential. Even though the International Metastatic RCC Database Consortium risk model is conventionally utilized for selection and stratification of patients with metastatic RCC (mRCC), there remains an unmet demand for novel prognostic and predictive markers. The goal of this study was to analyze the expression of Vascular endothelial growth factor (VEGF), Cluster of Differentiation 31 (CD31) to determine microvessel density, and Angiopoietin-1 (Ang-1) in primary kidney tumors, as well as their predictive and prognostic value in patients with metastatic ccRCC (mccRCC) who were treated with first-line sunitinib. The study included 35 mccRCC patients who were treated with first-line sunitinib in period between 2009 and 2019. Immunofluorescence was used to examine biomarker expression in tissue specimens of the primary tumor and surrounding normal kidney tissue. Median disease-free survival (DFS) was longer in patients with negative and low tumor VEGF score than in patients with medium tumor VEGF score (*p* ═ 0.02). Those with low tumor CD31 expression had a longer median DFS than patients with high tumor CD31 expression (*p* ═ 0.019). There was no correlation between Ang-1 expression and DFS. The expression of biomarkers in normal kidney tissue was significantly lower than in tumor tissue (*p* < 0.001). In conclusion, higher VEGF scores and greater CD31 expression were associated with longer DFS, but neither of these biomarkers correlated with progression-free survival or overall survival.

## Introduction

Renal cell carcinoma (RCC) occurs for 2%–3% of all malignancies in adults, with clear cell RCC (ccRCC) accounting for approximately 70% of cases [1]. Most patients with early-stage RCC can be treated surgically, but 33% of patients present in advanced stage of the disease when surgical treatment may not be curative [2]. Recent studies suggest the beneficial role of cytoreductive nephrectomy in patients with distant metastases [3]. In 50% of patients diagnosed with the early-stage disease who have undergone potentially curative surgical treatment, distant metastases can be expected during follow-up [4–7]. One of the distinguishing features of RCC is frequent metastatic spread [7–9].

RCC is very resistant to conventional chemotherapy, and patients with metastatic RCC (mRCC) were previously treated with cytokine-based therapy as a first-line treatment. The effectiveness of such therapy was limited, and mRCC had a poor prognosis. The role of the von Hippel-Lindau (VHL) gene in the pathogenesis of sporadic ccRCC has been recognized in molecular biology studies of the RCC. Namely, alterations in VHL lead to overexpression of Vascular Endothelial Growth Factor (VEGF) and several other growth factors, resulting in endothelial cell migration, tumor angiogenesis, and tumor growth [1, 10–13].

Tumor angiogenesis is a complex process involving cancer cells, tumor stroma cells, and vascular endothelial cells. The most important mediator of tumor angiogenesis is VEGF-A, which is normally expressed in the cytoplasm of tumor cells, endothelial cells, and stromal fibroblasts. In normal renal tissue, VEGF expression is limited to the cytoplasm of tubular epithelium, smooth muscle cells and macrophages in the interstitial space, as well as mesenteric cells in glomeruli [14]. Angiopoietins, which are ligands of Tie-2 Tyrosine Kinase Receptor (RTK) expressed primarily on endothelial cells, are also key components of tumor angiogenesis [15]. Cluster of differentiation 31 (CD31), also known as Platelet Endothelial Adhesion Molecule 1, is a 130 kDa glycoprotein expressed on endothelial cells, platelets, and some leukocytes. It is primarily used to detect the presence of endothelial cells by immunohistochemistry, which helps in determining the degree of tumor angiogenesis [16].

In the new era of targeted therapies, several drugs acting on the VEGF and mammalian Target of Rapamycin (mTOR) pathways have been developed. These medications are used as first- and second-line treatments for metastatic diseases, and they have been shown to considerably improve progression-free survival (PFS) and overall survival (OS) [7, 17, 18]. Sunitinib, a multi-targeted tyrosine kinase inhibitor (TKI) that targets VEGF and its receptors, is one of these medications. Sunitinib was shown to have a favorable impact in a phase III clinical study with 750 mccRCC patients who had a good/intermediate prognosis and had not previously received systemic therapy [18]. Until 2005, the only available therapy with a proven impact in a limited number of patients was cytokine therapy (interferon-alpha and high-dose interleukin-2). These drugs were gradually replaced by VEGF inhibitors, mTOR blockers, and, more recently, immune checkpoint inhibitors, which are not approved for the treatment of this condition in the Federation of Bosnia and Herzegovina (FBiH) [19].

Despite the fact that aforementioned targeted drugs dominate the therapeutic selection for advanced RCC, treatment decisions are made entirely on the basis of clinical criteria, without predictive and prognostic biomarkers. The International Metastatic RCC Database Consortium (IMDC) model, which integrates performance status (PS) and biochemical indicators, is one of the used prognostic models. The IMDC model was developed in the timeframe of targeted VEGF therapy and validated in a cohort of 1028 patients from 13 international centers. Each prognostic group, that is, favorable, intermediate, and poor, was associated with the median of the OS [20, 21].

Given the several available therapy options, validated predictive and prognostic biomarkers might be a useful guide in selecting suitable personalized treatments for patients with mRCC [22]. Patients in developing and low-income countries, on the other hand, have limited access to targeted therapies. Sunitinib has been available in FBIH since 2008, but there is a waiting list for this medicine, and treatment is usually delayed for several months once the drug is prescribed. Postponed treatment with targeted therapy significantly negatively affects survival, as well as cytogenetic and molecular response [23].

The extent of scientific research on prognostic and predictive markers in mRCC that might be useful in determining the best treatment strategy has dramatically increased in the last decade. The results of the previous studies are contradictory, and some of them included participants who had already been treated with other therapeutic options (interleukin, interferon-alpha, and chemotherapy) before starting sunitinib therapy. We found no relevant research papers in the literature that investigated the expression of CD31, VEGF, and Angiopoietin-1 (Ang-1) using immunofluorescence, their interconnection, and prognostic and predictive roles in patients with mccRCC treated with first-line sunitinib.

The goal of this study was to analyze the expression and coexpression of VEGF, CD31, and Ang-1 in the primary tumor and normal tissue, as well as their predictive and prognostic significance in patients with metastatic disease treated with sunitinib.

## Materials and methods

### Patients

A total of 55 mccRCC patients underwent a radical nephrectomy and were treated with sunitinib at the Oncology Clinic of University Clinical Hospital Mostar between 2009 and 2019. A total of 35 patients with available primary tumor tissue were enrolled in the study.

Patients with histopathological diagnosis of ccRCC, who presented with metastatic disease initially or during follow-up, and received sunitinib as a first-line treatment were eligible to participate in the research. We excluded patients who received prior targeted therapies, as well as patients with concurrent chronic renal disease which may impact the expression of the markers studied.

The clinical data were obtained from the hospital case records and patients were identified from the hospital registry and registry of patients treated with sunitinib obtained from Health Insurance and Reinsurance Institute of the FBiH (Solidarity Fund).

### Double immunofluorescence staining

Immunofluorescence was used to examine biomarker expression in histological specimens of the primary tumor and surrounding normal kidney tissue at the Laboratory of Morphology, Department of Histology and Embryology, School of Medicine, University of Mostar. After the histopathological diagnosis was confirmed, the samples were processed and stored in the archives of the Clinical Institute of Pathology, Cytology and Forensic Medicine of the University Clinical Hospital in Mostar, and Department of Pathological Anatomy and Cytology – Cantonal Hospital “Dr Safet Mujić.”

The process of double immunofluorescence staining was carried out according to the immunofluorescence protocols previously described [24].

The primary antibodies used were as follows: Anti- Angiopoietin 1 antibody, Goat polyclonal to Angiopoietin 1 (ab133425, abcam, United Kingdom, dilution 1:200); Anti-CD31 antibody [JC/70A]; Mouse monoclonal [JC/70A] to CD31 (ab9498, abcam, United Kingdom, dilution 1:2000); and Recombinant Anti-VEGFA antibody [EP1176Y] – C-terminal, Rabbit monoclonal [EP1176Y] to VEGFA – C-terminal, (ab52917, abcam, United Kingdom, dilution 1:250).

We employed immunofluorescence for secondary detection of the primary antibodies after incubation with primary antibodies and washing in PBS. In this method, to show the binding of the primary antibodies, the secondary antibodies of the animal origin of the primary antibodies used were as follows: Donkey Anti-Goat IgG H&L (Alexa Fluor^®^ 647) preadsorbed (ab150135, abcam, United Kingdom, dilution 1:500); Goat Anti-Mouse IgG H&L (Alexa Fluor^®^ 488) (ab150113, abcam, United Kingdom, dilution 1:1000); and Goat Anti-Rabbit IgG H&L (Alexa Fluor^®^ 594) preadsorbed (ab150084, abcam, United Kingdom, dilution 1:1000).

After washing with PBS, incubation with DAPI for 1 minute followed. The sections were washed in PBS once more before being embedded in the mounting media (Immuno Mount, Shandom, Pittsburg, PA, USA) and covered with coverslips. We analyzed the expression of applied markers in tissue. All tissue sections were inspected using a ×40 objective on Olympus BX51 microscope (Olympus, Tokyo, Japan) and photographed with DP71 digital camera (Olympus, Tokyo, Japan) attached to the microscope. Five representative areas from each section were used for analysis. For further image analysis, we used ImageJ software (National Institutes of Health, Bethesda, MD, USA), QuPath [25] and AdobePhotoshop (Adobe, San Jose, CA, USA).

In the case of immunofluorescence staining of each antigen, sections intended for negative control underwent the same procedure as other sections, with the exception that they were not incubated with the primary antibody and instead remained in PBS throughout that period. Only DAPI blue stained cell nuclei without fluorescent signal to the cytoplasm or cell nuclei were present in the negative control. The fluorescent signal (staining with fluorescent secondary antibodies) of individual cytoplasms or cell nuclei in the surrounding structures, which are known to react with primary antibodies, served as a positive control on the investigated sections. In addition, for each required factor, the distributions of the difference between positive and negative cells in the sections were examined in the literature. Two independent observers analyzed all sections separately, in a blinded manner, and all disagreements in interpretation were handled by consensus.

### Semi-quantification and quantification of biomarkers

Biomarker scoring was carried out using previously published and defined methods [26–30]. Because CD31 is an endothelial vessel marker, its expression was determined through the microvessel density (MVD) which was analyzed by counting individual CD31-stained microvessels in five fields at a magnification of 400 in a highly vascular tumor location (hot spot), omitting areas with extensive hyalinization and necrosis, as per consensus recommendations. A microvessel was defined as a CD31-positive endothelium or endothelial cell cluster with or without a viable lumen. In tumors with a dense microvasculature network, each branch was treated as a separate vessel. Single vessels were counted as large anastomosing sinusoidal vessels. Only vessels that were separate from one another were counted. The counting of large vessels with strong muscular walls was prohibited. The MVD value, which is a number without a unit, was calculated for each tumor based on the mean number of microvessels detected in five fields at ×400 magnification. The results were then scored on a scale from 1–3: score 1 (MVD ═ 1–49), score 2 (MVD ═ 50–100), score 3 (MVD > 100). CD31 in normal kidney tissue was examined according to the number of stained blood vessels on a scale from 1–3: 1 (0–10 blood vessels), 2 (11–20 blood vessels), and 3 (>20 blood vessels).

The percent of VEGF-stained cells in tumor and surrounding normal kidney tissue on five fields at ×400 magnification was assessed. The staining was graded on a 0 to four-point arbitrary scale for the percentage of positive cells: 0 ═ negative, 1 ═ <10%, 2 ═ 11%–50%, 3 ═ 51%–75%, and 4 ═ >75%. VEGF staining intensity was also graded using the following scale: 0-negative, 1-weak, 2-intermediate, and 3-strong.

The H-score was calculated by multiplying the percentage and intensity of VEGF staining using the formula: H score ═ % of VEGF-stained positive cells multiplied with intensity. The H-score ranged from 0–300, with 300 equaling 100% of cells stained strongly (3+). Furthermore, on a scale of 0–12, the grades of multiplied percentage of positive cells (0–4) and grade of intensity were divided into four groups: Negative (0–1), weak (2–5), medium (5–9), and strong (10–12) expression.

Endothelial VEGF was only determined by coexpression with CD31 positive blood vessels in tumor and normal surrounding kidney tissue. The results were graded on a0 scale of 0–3 as follows: 0-no staining, 1-partial coexpression, and 2-complete coexpression (Figure 2).

Ang-1 was determined by coexpression with CD31 positive blood vessels in tumor and normal kidney tissue as well, on a scale of 0–3: 0-no staining, 1-partial staining of blood vessels, and 2-complete staining of blood vessels.

### The assessment of prognosis and prediction of treatment response

OS was determined by taking into account the period from the date of surgery to the date of death or the most recent follow-up. Disease-free survival (DFS) was calculated from the date of surgery to the date of distant metastasis, and PFS was calculated from the start of sunitinib therapy to the date of the progression or the most recent follow-up.

Patients self-administered once-daily oral sunitinib 50 mg on a 4-week treatment followed by a 2-week off regimen. Based on individual tolerability, dose reductions to 37.5 mg and 25 mg of sunitinib were permitted. A diagnostic workup was performed before the start of the treatment, and evaluation was conducted according to the institution’s procedures, usually after 2–3 therapeutic cycles. A competent physician and the multidisciplinary tumor board made decisions on treatment delay, treatment discontinuation, dose modification, and subsequent therapy.

The IMDC prognostic model was used for the assessment and a retrospective analysis of clinical prognostic factors, which included the following: Hemoglobin concentration (<lower normal limits), high corrected calcium (>upper normal limits), high neutrophil count (>upper normal limits), high platelet count (>upper normal limits), low Karnofsky PS (<80%), and time from diagnosis to treatment <1 year. Patients were divided into three categories based on the number of adverse factors: Favorable, intermediate, and poor risk group (0, 1–2, and 3–6, respectively).

### Ethical statement

The study was approved by the Institutional Ethics Committee (protocol number 245/21, approved February 22, 2021). The study was conducted in accordance with the guidelines of the Declaration of Helsinki. Before sunitinib treatment, all patients signed informed consent, in which they read and understood, and agreed that their data could be used for scientific purposes.

### Statistical analysis

The descriptive measures, such as absolute value and percentages, were defined. The Kaplan–Meier method with the log-rank test was used to investigate the relation between tumor CD31 and VEGF score with DFS. The Wilcoxon signed-rank test was used to compare relations of CD31, VEGF, and Ang-1 in tumor and normal kidney tissue. *p* < 0.05 was an indicator of significance. The statistical analysis was performed using IBM SPSS Statistics v. 23.0.

### Data availability

The datasets used and/or analyzed in the present study are available from the corresponding author on request.

## Results

The demographic and clinical characteristics of patients are presented in Table 1. There were 35 patients with mean age of 60.31 years ± 8.828. Median time from sunitinib prescription to treatment initiation was 2 months (range 0–8). Mean age at the start of sunitinib treatment was 61.29 ± 8.854. Median time from mRCC diagnosis to sunitinib initiation was 3 months (range 1–9).

Median DFS was 2 months (range 0–92), median first-line PFS was 8 months (range 1–116), and median OS was 29 months (range 2–116).

### Biomarker expression

#### Expression of VEGF

In tumor tissue, according to groupings of multiplied percentage of VEGF positive cells and grade of intensity, the VEGF negative score consisted of 3 (8.6%) tumors, the VEGF weak score of 9 (25.7%) tumors, and the VEGF medium score of 23 (65.7%) tumors.

In normal kidney tissue, of 20 controls, the VEGF negative score consisted of 6 (30%) controls and the VEGF weak score of 14 (70%) controls.

Median H score in tumor tissue was 50.256 (range 0.033–193.890), while in normal kidney tissue, it was 1.156 (range 0–74.354).

**Table 1:** Patients demographic and disease characteristics

**Variables**	**Number**	**Percent**
Gender		
Male	24	68.6
Female	11	31.4
Grade of tumor		
2	14	40.0
3	15	42.9
4	6	17.1
Tumor necrosis		
Not present	8	22.9
Mild	4	11.4
Present	9	25.7
Abundant	14	40.0
Tumor hemorrhage		
Not present	20	57.1
Mild	5	14.3
Present	4	11.4
Abundant	6	17.1
Capsule infiltration		
Yes	30	85.7
No	5	14.3
Pathological T		
1	9	25.7
2	2	5.7
3	21	60.0
4	3	8.6
Tumor stage		
1	9	25.7
2	2	5.7
3	20	57.1
4	4	11.4
Tumor laterality		
Right	14	40.0
Left	21	60.0
Metastatic disease presentation		
Synchronous	21	60.0
Metachronous	14	40.0
Presentation of metastatic disease - 1 year		
<1 year	28	80.0
>1 year	7	20.0
Site of metastasis		
Lung	26	74.3
Lymph node	4	11.4
Bone	10	28.6
Liver	5	14.3
Kidney	1	2.9
Brain	1	2.9
Other	5	14.3
Metastasis number		
Solitary	2	5.7
Multiple	33	94.3
Number of metastatic sites		
1	21	60.0
2	10	28.6
3	3	8.6
4	1	2.9
IMDC prognostic group		
Favorable	3	8.6
Intermediate	15	42.9
Poor	17	48.6
ECOG PS		
0	16	45.7
1	14	40.0
2	5	14.3
Presence of side effects	15	42.9
Dose reduction due to side effects	9	25.7

**Figure 1.**
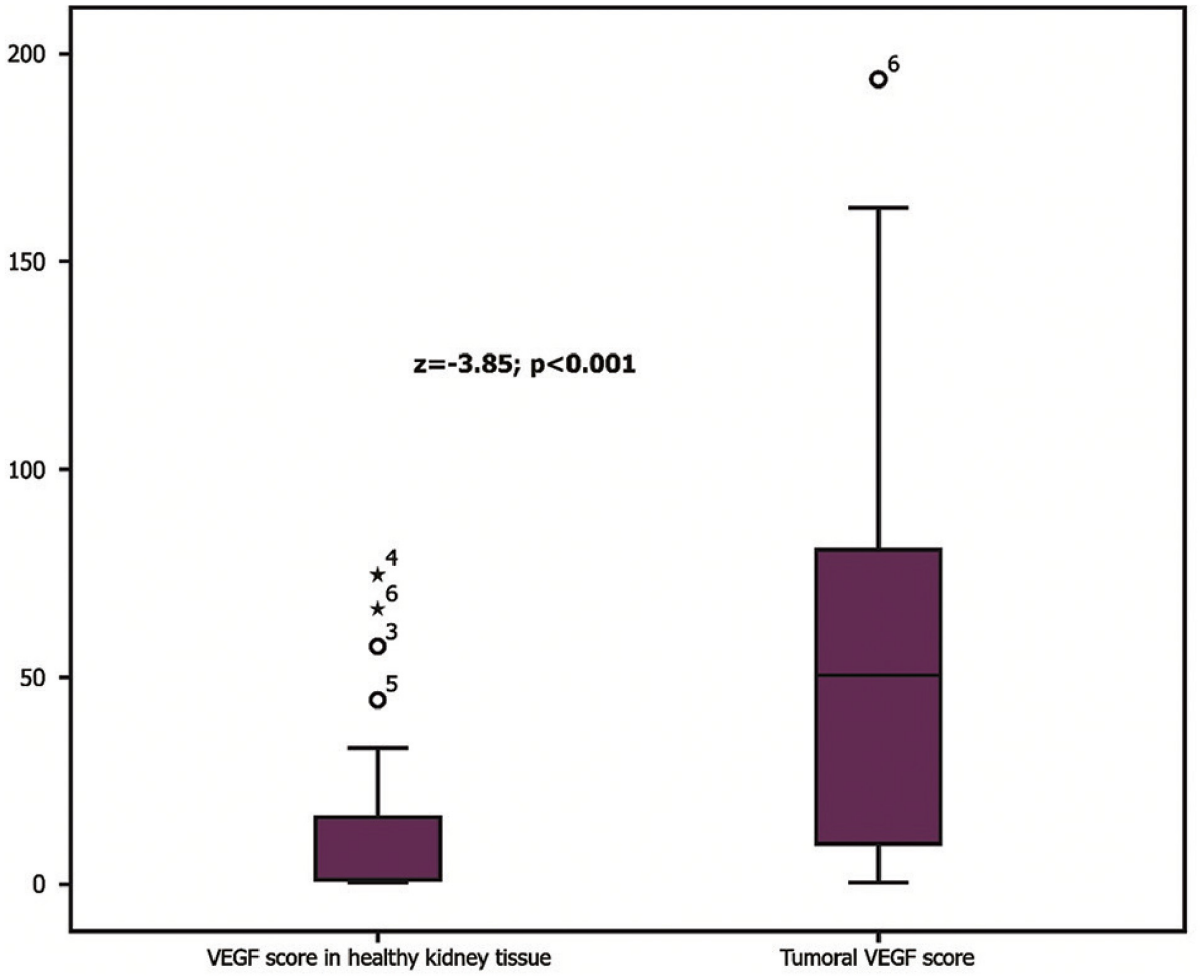
**VEGF (H) score in normal kidney tissue compared to tumor tissue.** Wilcoxon signed-rank test identified significantly lower values of VEGF (H) score in normal kidney tissue compared to tumor tissue, *z* ═−3.85, *p* < 0.001. Median VEGF score (interquartile range) in normal kidney tissue was 1.156 (0–16.644) and in tumor tissue 50.256 (8.949–84.315).

**Figure 2.**
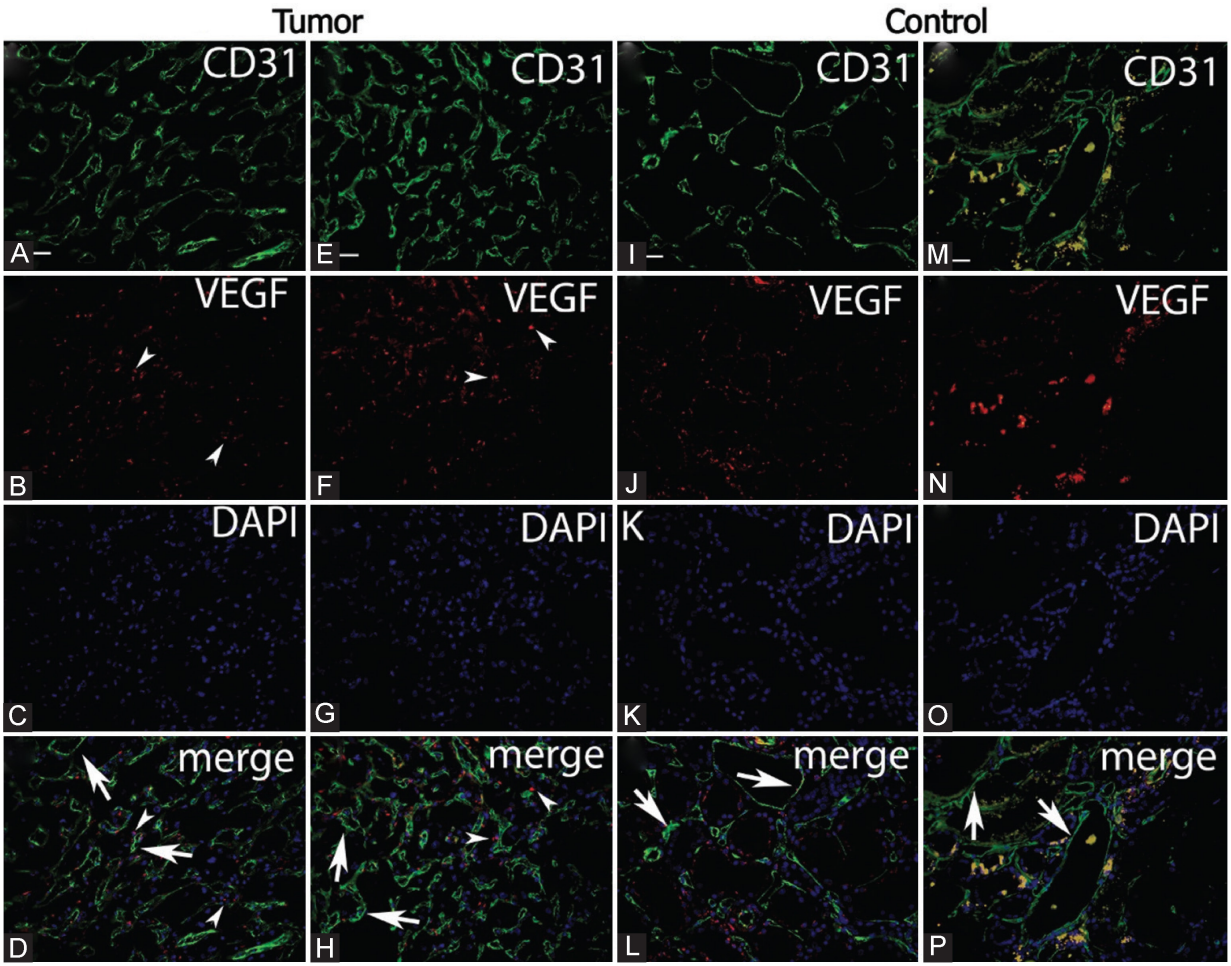
**Double immunofluorescence staining revealed no coexpression of CD31 and VEGF markers in blood vessels of renal cell carcinoma (arrows) (D and H).** Tumor VEGF H score in tumor tissue (B and F) was higher than in normal kidney tissue (J and N). Double immunofluorescence staining showed higher values of expression of CD31 stained blood vessels of tumor tissue (A and E) than in normal kidney tissue (I and M). Magnification ×400. Scale bar ═ 25 µm. (Wilcoxon signed-rank test, *p* < 0.001).

There were significantly lower values of VEGF (H) score in normal kidney tissue compared to tumor tissue (z ═ −3.85, *p* < 0.001). Normal kidney tissue had a median VEGF score (interquartile range) of 1.156 (0–16.644), while tumor tissue had a value of 50.256. (8.949–84.315). These findings are shown in Figures 1 and 2.

#### Expression of CD31

In tumor tissue, mean MVD was 66.85 ± 27.196. According to the 1–3 score, the number of patients with expression scores 1, 2, and 3 were 12 (34.29%), 19 (54.29%) and 4 (11.42%), respectively.

In normal kidney tissue of 20 controls, according to the 1–3 score, the number of patients with expression scores 1, 2, and 3 were 12 (60%), 7 (35%) and 1 (5%), respectively.

**Figure 3.**
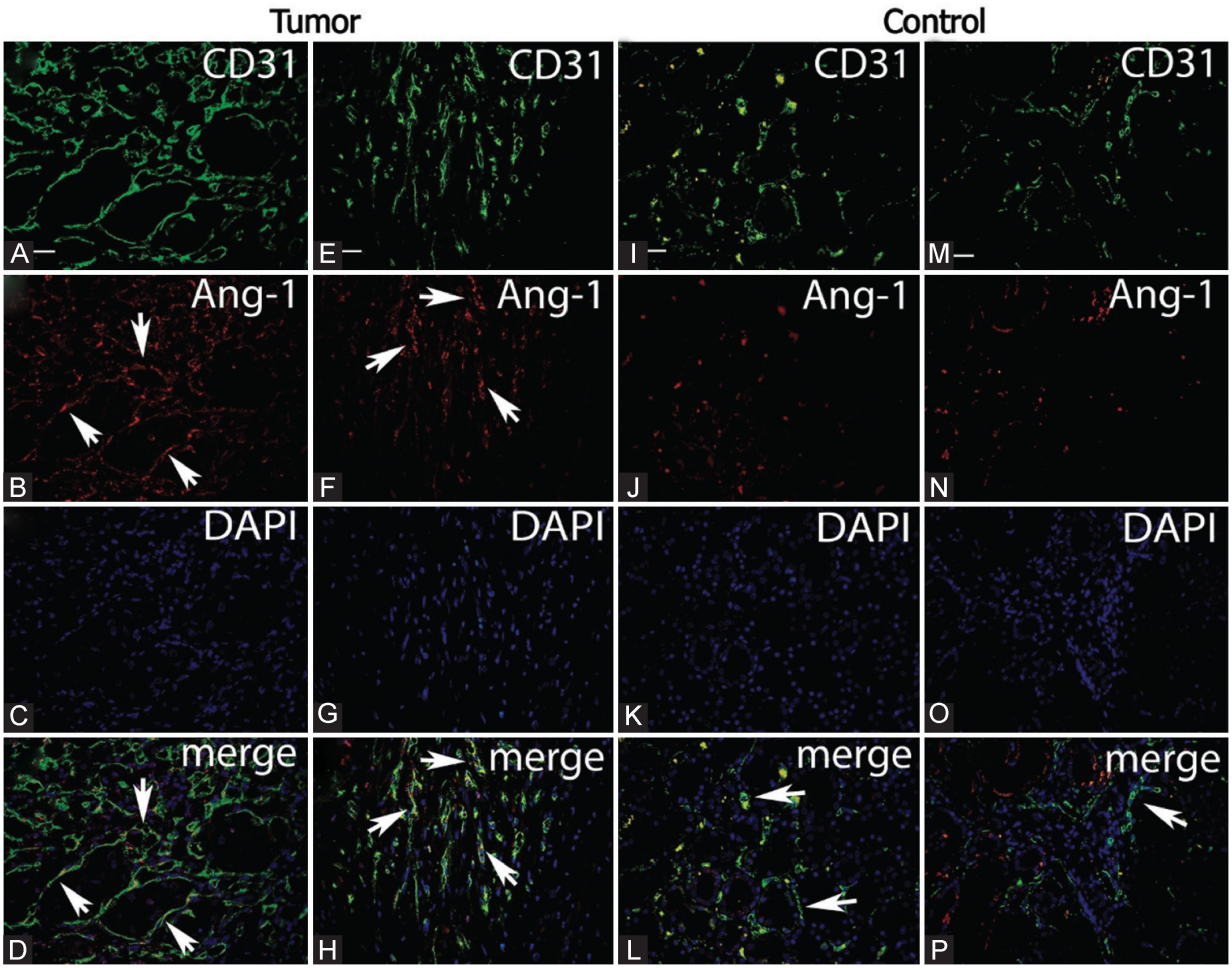
**Coexpression of CD31 and Ang-1 markers in blood vessels of renal cell carcinoma (A-H).** Double immunofluorescence staining showed higher expression of Ang-1 in blood vessels of tumor tissue (B and F). Merge A+B and E+F with DAPI showing coexpression of CD31 and Ang-1 (arrows) (D and H). Double immunofluorescence staining showed higher values of expression of CD31 stained blood vessels of tumor tissue (A and E) than in the normal kidney tissue (I and M). Magnification ×400. Scale bar ═ 25 µm. (Wilcoxon signed-rank test, *p* < 0.001).

Normal kidney tissue had significantly lower levels of CD31 expression than tumor tissue (*z* ═ −3.92, *p* < 0.001). In normal kidney tissue, the median CD31 score (interquartile range) was 1 (1–2), and in tumor tissue, it was 65 (43.0–90.5). These findings are shown in Figures 2 and 3.

#### Expression of Ang-1

In RCC tissue, there was negative coexpression of Ang-1 with CD31 (in CD31-stained blood vessels) in 2 (5.7%) patients, partial coexpression in 11 (31.4%) patients, and positive co-expression in 22 (62.9%) patients. In normal kidney tissue, of 20 controls, 17 (85%) patients had negative coexpression with CD31 and only 3 (15%) had partial coexpression (Figure 3).

Normal kidney tissue had significantly lower values of Ang-1 than tumor tissue (*z* ═ −3.84, *p* < 0.001). Normal kidney tissue had a median Ang-1 score (interquartile range) of 0 (0), while tumor tissue had a score of 2 (1–2). These findings are shown in Figure 3.

### Survival analysis

**Figure 4.**
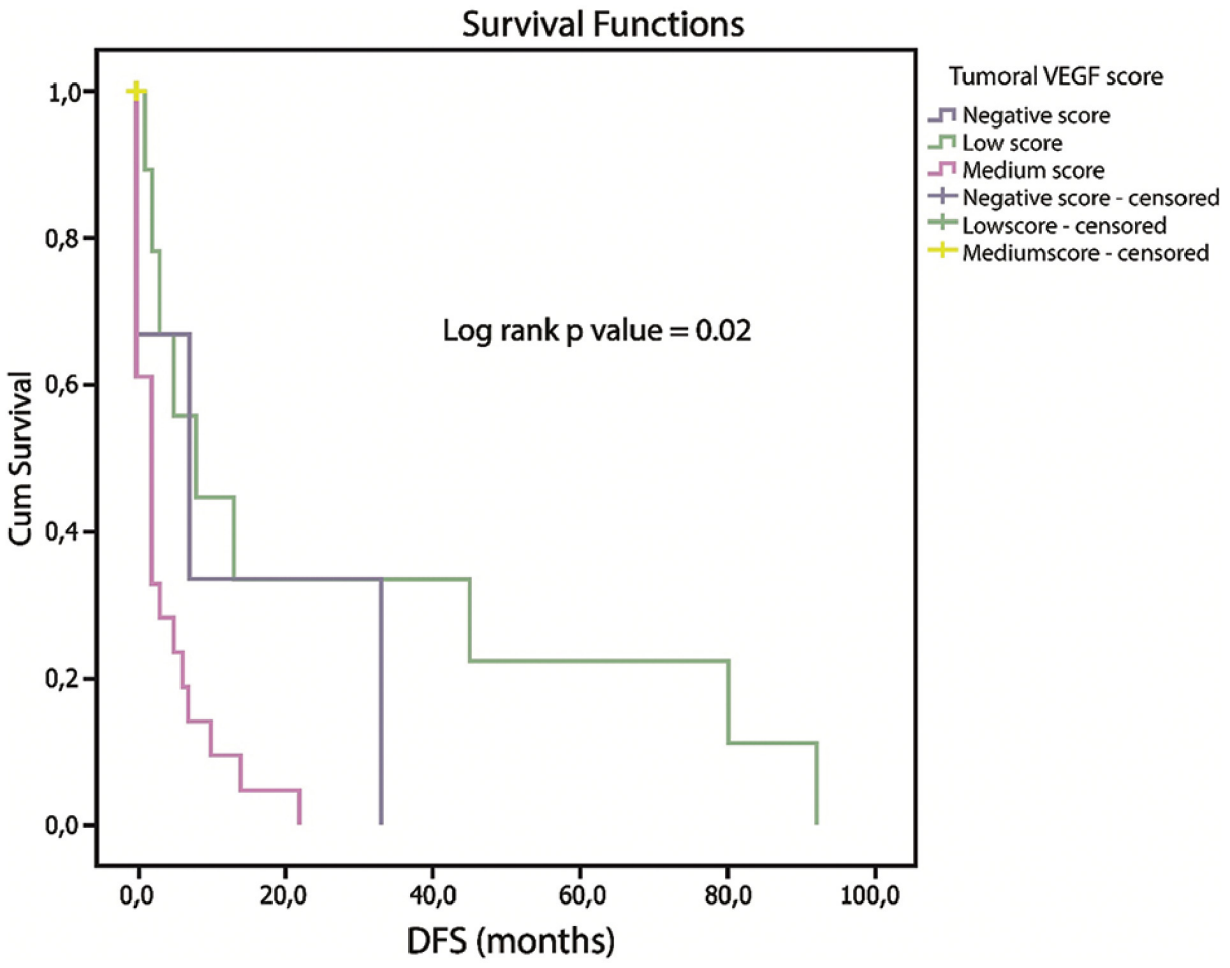
**Kaplan–Meier curves showing distant-free survival (DFS), stratified by tumor VEGF score.** Median DFS in patients with negative, low, and medium tumor VEGF score was 7.0 (0–33.0), 8.0 (1–92.0), and 2.0 (0–22.0) months, respectively (*p* ═ 0.02).

**Figure 5.**
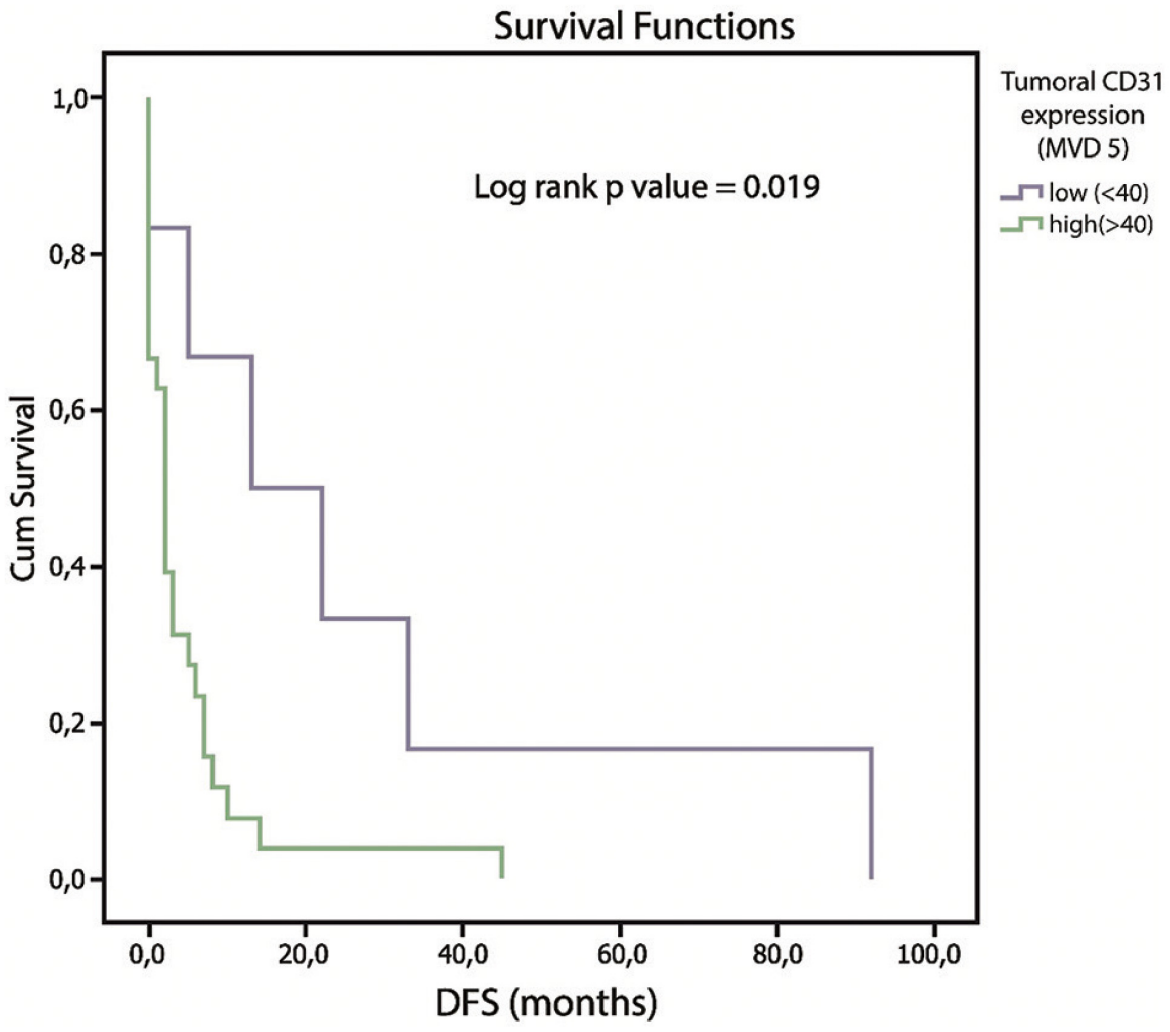
**Kaplan–Meier curves showing distant-free survival (DFS), stratified by tumor CD31 score.** Median DFS in patients with low and high tumor CD31 expression was 17.5 (0–92.0) and 2.0 (0–45.0) months (*p* ═ 0.019).

DFS was examined in relation to the cutoff value of tumor CD31 expression (cutoff value-40 microvessels) and tumor VEGF score of the tumor. Median DFS in patients with a negative VEGF score was 7.0 (0–33.0) months, 8.0 (1–92.0) months in patients with low VEGF score, and 2.0 (0–22.0) months in patients with medium VEGF score (*p* ═ 0.02) (Figure 4). There were no patients with a high VEGF score in our research sample. Patients with low CD31 expression had a median DFS of 17.5 (0–92.0) months, whereas those with high CD31 expression had a median DFS of 2.0 (0–45.0) months (*p* ═ 0.019) (Figure 5).

## Discussion

Traditional prognostic factors are helpful in RCC, but they cannot entirely predict the outcome for each patient. Despite the results of previous research, there is still a shortage of reliable biomarkers for this disease, which is critical in the era of novel targeted therapies as well as immunotherapies.

We analyzed the expression of an angiogenic biomarker in archival tissue from patients with mccRCC who received first-line treatment with sunitinib. In comparison to normal kidney samples, we found higher expression of VEGF, CD31, and Ang-1 biomarkers in tumor samples.

In terms of VEGF expression, the findings are consistent with prior reports in the literature. The results of the research by Veselaj et al. demonstrated a higher expression of VEGF in tumor tissue in RCC patients than in normal kidney tissue, supported by our findings [31]. In research conducted by Nicol et al., results revealed VEGF overexpression in RCC but not in normal renal tissue [32]. Different techniques (RT-PCR and Western blot analysis) were utilized as research methods in this study, which might explain some of the findings. To shed light on this, other research with additional samples are required.

Because ccRCC is a highly vascularized solid tumor type [33], it was hypothesized that the MVD and VEGF expression in tumor tissue might be employed as a prognostic and subsequently as a predictive factor for sunitinib therapy, which served as the foundation for many investigations in this field.

Our findings revealed that patients with low CD31 expression in tumor tissue had a longer median DFS than those with high CD31 expression. Furthermore, patients with a lower VEGF score had a longer median DFS than those with a moderate VEGF score, which is consistent with the findings of Minardi et al., who pointed out that VEGF expression was associated with DFS and OS, but not with response to sunitinib treatment [34]. These findings suggest that angiogenic activity may be increased in tumors with a higher potential to invade, which supports the results of prior studies that RCC has a higher production of VEGF as well as a tendency to metastasize.

We discovered no significant association between biomarker expression and responsiveness to sunitinib treatment in our data, however, unlike Minardi et al. and Liontos et al. [27], we found no association between biomarkers and OS.

There have been many discrepancies in previous studies regarding the expression of VEGF and CD31, as well as their prognostic and predictive roles in mRCC.

In contrast to the results of our study and that of Minardi et al., Trávníček et al. reported in 2017 that significantly higher expression of VEGF in ccRCC than in normal parenchyma might indicate a better response to sunitinib [35].

Our findings revealed a higher expression of CD31 in tumor tissue than in normal kidney tissue, as well as a longer median DFS in patients with lower CD31 expression.

MVD has been linked to advanced pathological features and poor clinical prognosis in renal cancer patients, and metastases are more likely in those with highly vascularized tumors, suggesting that tumor vascularization may be linked to disease outcome. When compared to normal tissue vasculature, tumor-associated vasculature has increased MVD values. Despite multiple studies demonstrating MVD’s clinical prognostic significance in other types of tumors, its prognostic value for RCC outcomes is still controversial [36].

According to Rautiola et al. in 2016, higher Ang-2 expression in the primary tumor was associated with clinical benefit rate but not with PFS and OS in patients with mRCC treated with first-line sunitinib, while low Ki-67 was associated with longer PFS and OS [29].

This is one of the few studies, in which Ang-2 levels have been determined in tumor tissue rather than in patient serum. The expression of endothelial Ang-2 in the tumor vasculature was found to be linked to tumor vascular density, as determined by CD31 expression. In our study, Ang-1 was only found in CD31 marker blood vessels in tumor tissue, and its expression did not significantly correlate with OS or responsiveness to sunitinib therapy.

In 2013, Dornbusch et al. discovered that immunohistochemical expression of CD31, pVEGFR1 and VEGFR1 and -2 in the primary tumor may be a predictor of a good response to sunitinib in mRCC patients treated with this medicine [37]. Other studies have investigated the serum VEGF levels of subjects with mRCC and the association with the response to sunitinib therapy [38]. Furthermore, serum levels of Ang-1 and -2 were examined in most studies, rather than their tumor expression [39].

New attempts to create validated biomarkers to identify patients with mRCC for suitable therapy include so-called gene signatures, tumor gene expression, and immune cells analyses [39–43]. In one of these studies, there was no significant difference in the angiogenesis gene profile between three different IMDC risk groups, which are in correlation with our findings and suggests that the prediction of an enhanced TKI response is independent of previously established clinical prognostic markers [41].

According to several research, enhanced regulation of a set of genes linked to angiogenesis might predict a better response to anti-VEGF therapy. Participants with low angiogenesis gene expression did not benefit as much from this therapy, possibly due to an immunologically enriched tumor subtype that is more likely to respond to ICIs therapy [22].

As per the literature, this is the first study to use an immunofluorescence approach to investigate angiogenic biomarkers in patients with mccRCC treated with first-line sunitinib, whereas in other studies, patients received different therapy prior sunitinib. Given the study’s limitations (sample size, single-center study), further research with a larger number of patients is needed to investigate the role of these biomarkers in mccRCC, and to corroborate earlier findings to validate potential prognostic and predictive biomarkers.

## Conclusion

Finally, our findings suggest that CD31 and VEGF expression in tumor tissue might be used as prognostic markers for DFS in mccRCC patients. Given the study’s limitations, the analyzed biomarkers VEGF, CD31, and Ang-1 do not appear to be promising as indicators of therapeutic response to first- line sunitinib therapy. However, further large-scale controlled prospective trials are required.

Today, combined treatment with antiangiogenic drugs and ICIs is the therapeutic standard for most patients with mRCC. Therefore, it would be practically worth knowing whether angiogenesis-related markers or immune markers might assist in predicting the therapeutic response, to reduce toxicity while also optimizing treatment efficacy. This might serve as a starting point for future studies.

## Acknowledgments

The authors thank the staff of Laboratory of Morphology, Department of Histology and Embryology, School of Medicine, University of Mostar; Department of Pathology, Cytology and Forensic medicine, University Clinical Hospital Mostar; Department of Pathological Anatomy and Cytology, County Hospital “Dr Safet Mujič” (Mostar, Bosnia and Herzegovina); and Department of Clinical Pathology and Cytology, Clinical Center of the University of Sarajevo (Sarajevo, Bosnia and Herzegovina), Health Insurance and Reinsurance Institute of the Federation of BiH (Solidarity Fund). We particularly acknowledge Prof. Katarina Vukojević, Prof. Violeta Šoljić, Prof. Nurija Bilalović and Prof. Amina Kurtović-Kozarić for all support.

**Conflicts of interest:** The authors declare no conflicts of interest.

**Funding:** The project was implemented with the financial support of the Central State Office for Croats Outside the Republic of Croatia (“Središnji državni ured za Hrvate izvan Republike Hrvatske”).
